# Biomechanical and functional indicators in male semiprofessional soccer players with increased hip alpha angles vs. amateur soccer players

**DOI:** 10.1186/1471-2474-15-88

**Published:** 2014-03-16

**Authors:** Matthias Lahner, Christoph von Schulze Pellengahr, Philipp Alexander Walter, Carsten Lukas, Andreas Falarzik, Kiriakos Daniilidis, Lars Victor von Engelhardt, Christoph Abraham, Ewald M Hennig, Marco Hagen

**Affiliations:** 1 Department of Orthopaedic Surgery, Ruhr-University Bochum, St. Josef-Hospital, Gudrunstr. 56, 44791 Bochum, Germany; 2 Department of Diagnostic and Interventional Radiology and Nuclear Medicine, Ruhr-University Bochum, St. Josef-Hospital, Gudrunstr. 56, 44791 Bochum, Germany; 3 Olympic Training Center Westfalen/Bochum, Hollandstr. 95, 44866 Bochum, Germany; 4 Department of Orthopaedic Surgery, Annastift Hannover, (Medical School Hannover, MHH), Anna-von-Borries-Str. 1-7, 30625 Hannover, Germany; 5 Faculty of Health Sciences, University of Witten/Herdecke, Alfred-Herrhausen-Str. 50, 58448 Witten, Germany; 6 Biomechanics Laboratory, Department of Sport and Movement Sciences, University of Duisburg-Essen, Gladbecker Str. 182, 45141 Essen, Germany

**Keywords:** Rearfoot motion, Tibial acceleration, Alpha angle of Nötzli, Femoroacetabular impingement, Magnetic resonance imaging, Soccer players

## Abstract

**Background:**

Femoroacetabular impingement (FAI) is predominant in young male athletes, but not much is known about gait differences in cases of increased hip alpha angles. In our study, the hip alpha angle of Nötzli of soccer players was quantified on the basis of magnetic resonance imaging (MRI) with axial oblique sequences. The aim of the current study was to compare the rearfoot motion and plantar pressure in male semiprofessional soccer players with increased alpha angles to age-matched amateur soccer players.

**Methods:**

In a prospective analysis, male semiprofessional and amateur soccer players had an MRI of the right hip to measure the alpha angle of Nötzli. In a biomechanical laboratory setting, 14 of these participants in each group ran in two shoe conditions. Simultaneously in-shoe pressure distribution, tibial acceleration, and rearfoot motion measurements of the right foot were performed.

**Results:**

In the semiprofessional soccer group, the mean value of the alpha angle of group was 55.1 ± 6.58° (range 43.2-76.6°) and 51.6 ± 4.43° (range 41.9-58.8°) in the amateur group. In both shoe conditions, we found a significant difference between the two groups concerning the ground reaction forces, tibial acceleration, rearfoot motion and plantar pressure parameters (*P* < 0.01, *P* < 0.05, *P* = 0.04). Maximum rearfoot motion is about 22% lower in the semiprofessional group compared to the amateur group in both shoe conditions.

**Conclusions:**

This study confirmed that semiprofessional soccer players with increased alpha angles showed differences in gait kinematics compared to the amateur group. These findings support the need for a screening program for competitive soccer players. In cases of a conspicuous gait analysis and symptomatic hip pain, FAI must be ruled out by further diagnostic tests.

## Background

Femoroacetabular impingement (FAI) is a morphological hip disorder which shows a novel approximation to mechanical etiology of hip osteoarthritis [1,2]. A large share of idiopathic hip arthritis can be attributed to FAI, which is why early diagnosis is very important [3,4]. In the pathogenesis of FAI, there are two anatomical deformities either at the acetabulum or the proximal end of the femur or in both structures. A femoral type (cam impingement) is anatomically differentiated from the acetabular type of FAI (pincer impingement) [5]. The cam impingement is caused from a prominence at the anterolateral femoral head-neck junction [6]. The alpha angle of Nötzli is described to quantify the asphericity of the femoral head in axial oblique sequences of magnetic resonance images (MRI). Causes for the cam impingement are aspheric deformity of the femoral head, slipped capital femoral epiphysis, late closure of the femoral epiphysis and Legg-Calve-Perthes disease [7-9]. The pincer impingement is caused by an immoderate acetabular cover of the head of femur and is linked with acetabular retroversion, protrusio acetabuli or coxa profunda [10,11]. Symptomatic FAI can be treated by arthroscopic procedures or open surgery [12].

FAI is assumed to be predominant in young male athletes with sport activities with high impact for the hip joints like soccer [13]. Agricola et al. demonstrated that FAI was more prevalent in 89 elite soccer players than in 92 nonathletic controls [14]. Cam-type deformity develops during adolescence and is probably to be affected by high-impact sports practice [14]. The soccer game is primarily characterized by running-related actions [15]. Therefore, the shock attenuation capacity of soccer players with FAI during running is of special biomechanical interest.

Clinically, FAI must be differentiated from insertional tendinopathy of the adductor muscles. The tendinopathy of the adductor can be associated with arthropathy of the symphysis and insertional pubic area [16]. The major clinical symptoms of the tendinopathy of the adductor muscles are groin or lower abdomen pain [16].

Only few biomechanical studies exist on the gait analyses in patients with FAI [17-20]. In these studies, symptomatic patients were compared to healthy control probands. However, to our best knowledge, this is the first study which analyzed a strongly selective risk group like male semiprofessional soccer players who are disproportionately affected by FAI, but usually do not show clinical symptoms, yet.

Therefore, the aim of our biomechanical study was to compare the foot rollover process during running between male semiprofessional soccer players with increased alpha angles and age-matched amateur soccer players. It was postulated that an increased alpha angle would lead to different rearfoot motion, tibial acceleration and plantar pressure parameters.

## Methods

### Study participants

This study follows the Declaration of Helsinki. All probands have voluntarily agreed to the study and gave their informed consent. All study persons have received and signed patient education for MRI. The study protocol was approved by the local ethical committee of the Ruhr-University Bochum (registration number 4370-12). Between January 2012 and July 2013, 14 male semiprofessional soccer players and 14 male amateur soccer players underwent a clinical examination of the mechanical leg axis, an MRI and a gait analysis. The MRI data was published in a previous study [21]. The soccer players were semiprofessional athletes who played 4 training units per week for 2 hours with a seasonal duration of 10 months. The control group consisted of age-matched male amateur soccer players with a physical activity less than 5 hours a week. In all cases, the right leg was the kicking leg. For both groups, exclusion criteria were any kind of previous hip surgery in either hip joint, inflammatory or metabolic rheumatic disease or a history of haemophilia. Height and weight measurements were performed. Dorsal or knee pain was excluded by the clinical examination.

### MRI protocol

Each study volunteer underwent a nonarthrogram 1.5-T MRI (Magnetom, Siemens, Erlangen, Germany) of the right hip. All patients were examined in the supine position with a neutral position of the hip joint. The MRI sequences were obtained on each study test person by one specially-trained radiology technician. The MRI sequences parameters of the turbo spin-echo sequence were as follows: repetition times (TR) 637 ms, echo time (TE) 14 ms, a field of view (FOV) of 350 × 350 mm, a matrix of 512 × 256, a slice thickness of 6 mm and flip angle 150°. In addition, we used a coronal T1-weighted sequence (TR 530 ms, TE 14 ms, FOV 400 × 400 mm, slice thickness 5 mm, flip angle 150°), axial oblique T1-weighted sequence (TR 530 ms, TE 14 ms, FO 350 × 265 mm, slice thickness 5 mm, flip angle 150°) oriented along the axis of the femoral neck and fat-suppressed T1-weighted fast low angle shot (FLASH) sequences (TR 795 ms, TE 11 ms, FOV 400 × 400 mm, slice thickness 3 mm, flip angle 60°). For ethical reasons, neither intraarticular nor intravenous contrast was injected. The alpha angle was analyzed using the technique described by Nötzli [6]. We considered an alpha angle >55° as cut-off value because an alpha angle >55° is associated with FAI [22]. The alpha angle was subsequently measured by a radiologist (C.L.) and an orthopaedic surgeon (M.L.) both experienced in musculoskeletal imaging. The radiologist was blinded to the level of activity of the subjects.

### Biomechanical measurements

In a biomechanical laboratory setting, each participant of both groups ran in two shoe conditions (NW: regular running shoe; Crane, Isa Traesko, Germany; VW: same shoe with inserted valgus wedges, mediolateral height difference: 1 cm) at a speed of 3.3 m · s^-1^ across a piezoelectric force platform (Kistler 9281 B). Running speed was controlled by two photocells at equal distances in front of and behind the force platform. Only running trials within ±3% of the target speed were accepted. Five successful trials were recorded in each condition. Simultaneously in-shoe pressure distribution, tibial acceleration, and rearfoot motion measurements of the right foot were performed. Seven anatomical locations of the foot (medial and lateral heel; lateral midfoot; first, third and fifth metatarsal heads; hallux) were palpated, and piezoceramic transducers (4×4×2 mm; Halm, Germany) were fastened under the foot with adhesive tape. The physical properties of the piezoceramic transducers were described by Hennig et al. [23]. To measure tibial acceleration, an Entran EGAX-F-25 miniature accelerometer was glued to the skin above the medial aspect of the tibia at a location midway between medial malleolus and tibial plateau. The accelerometer was fastened by an elastic strapt to improve the mechanical coupling to the underlying bone. Rearfoot motion was measured by using an electrogoniometer (Megatron MP 10) that was attached to the heel counter of the shoe. Rearfoot angle was defined as the angle between rearfoot bisection and the direction of the achilles tendon. The detailed biomechanical setup was presented in the study of Milani et al. [24].

### Data collection and processing

The ground reaction force, axial tibial acceleration, pressure distribution, and rearfoot motion were collected simultaneously by a computer in a pretrigger mode. The data were sampled at a rate of 1 kHz per channel with a resolution of 12 bits. A threshold of 5 N of the vertical ground reaction force was chosen to determine the time onset of foot strike. The force and acceleration values were determined as multiples of body weight (bw) and gravitational acceleration (g), respectively. The maximum force rate was calculated as the highest differential quotient of adjoining vertical ground reaction force divided by the time resolution of 1 ms. The median power frequency of the vertical force signal was calculated from a 1,024-point FFT power spectrum analysis. A low frequency cut-off value of 10 Hz was chosen, since only the initial impact force was of interest. Under the seven anatomical locations, peak pressures were determined for all participants. Maximum range of rearfoot motion was chosen as a descriptor value for the pronation behaviour of the foot.

### Statistical analysis

The selected patient cohort was grouped in an Excel file (version 2003, Microsoft Corporation, Seattle, USA). Distribution of data was assessed by the D’Agostino-Pearson test. The arithmetic mean value, SD and 95% confidence intervals were calculated for the variables above and measured with Microsoft Excel. The values were recorded in IBM SPSS Statistics 14 (PASW 14, SPSS Inc., Chicago, IL, USA). The measurements on the alpha angles was compared using the Student’s *t*-test. Inter-rater reliability was measured of the MRI readings. Statistical significance was defined as a P value <0.05.

## Results

### MRI

**Figure 1:**
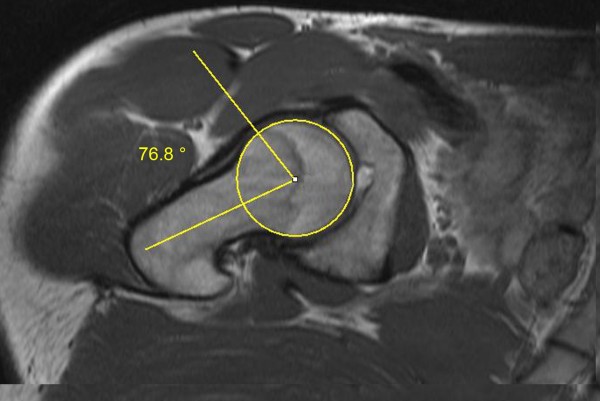
**Axial MRI scan of the right hip of a participant of the soccer group.** The alpha angle is formed by the connecting lines (yellow lines) between the longitudinal mid-axis of the femoral neck and the axis which marked the point first exceeded the radius of the cartilage-covered femoral head. The alpha angle was 76.8° in this example.

**Figure 2:**
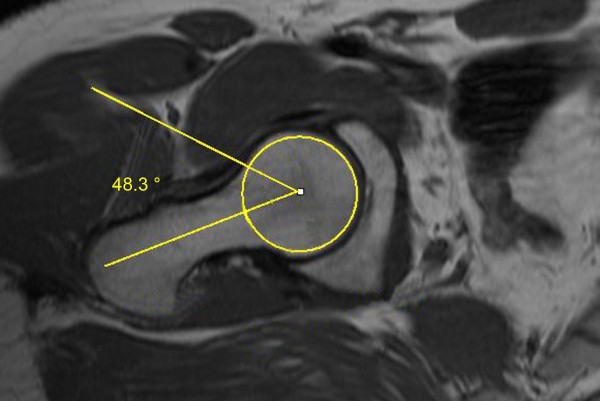
**Axial MRI sequence of a participant of the amateur group.** The alpha angle of the right hip was 48.3°.

The demographic data is presented in Table 1. There was no statistically significant difference concerning the mean age of both groups. There was no statistically significance difference in height, weight and body-mass index. 7 (50%) subjects of the semiprofessional group and 5 (35.7%) subjects of the amateur group clinically showed a varus malalignement of the mechanical axis. In both groups, no one had a history of hip dysplasia or disease in the childhood. In the soccer group, the mean value of the alpha angle was 55.16 ± 6.58° (Figure 1). The mean value of the alpha angle was lower in the amateur group, but there was no significant difference in the amateur group (51.65 ± 4.43°, Table 2, Figure 2)*.* Concerning the inter-rater reliability, the values ranged from 0.87 to 0.99 (Table 3).

**Table 1:** Demographic information

**Group (n)**	**Age (years)**	**Height (cm)**	**Weight (kg)**	**Body mass index (kg/cm**^**2**^**)**
Semiprofessional group (14)	22.21 ± 2.28	180 ± 0.07	76.0 ± 9.18	23.2 ± 1.25
Amateur group (14)	22.71 ± 2.88	181 ± 0.07	81.28 ± 11.11	23.80 ± 3.03
*P* value	0.496	0.722	0.182	0.500

**Table 2:** Comparison of the mean of the alpha angles of the two groups

**Group division (n)**	**Mean alpha angle ± SD**
Semiprofessional group (14)	55.16 ± 6.58°
Amateur group (14)	51.65 ± 4.43°
*P* value	0.11

**Table 3:** Inter-rater reliability of the alpha angles between the two observers

**Variables**	**Inter-rater reliability**	**95%-confidence interval**	
Oberserver 1 vs. observer 2		Minimum level	Maximum level
Alpha angle, semiprofessional group	0.966	0.87	0.99
Alpha angle, amateur group	0.967	0.899	0.989

### Biomechanical measurements

The results (mean values and standard deviations) of the ground reaction forces, peak tibial acceleration, rearfoot motion and plantar pressure parameters are presented in Tables 4 and 5. In both shoe conditions, we found similar statistically significant differences between the groups. Increases in median power frequency (NW: +7%, p < 0.05, VW: +8%, p < 0.05) and the rate of the vertical ground reaction force (NW: +39%, p < 0.01, VW: +56%, p < 0.05) reveal higher loading of the lower extremities and shock transmission to the upper body in the semiprofessional subjects.

**Table 4:** NW shod running without inserted valgus wedges

**Variables**	**Semiprofessional group mean**	**SD**	**Amateur group mean**	**SD**	***P *** **value**
Loading rate (bw/s)	101.9	32.2	73.2	8.6	<0.01**
Peak tibial acceleration (g)	7.8	2.3	5.9	2.6	<0.05*
Median power frequency (Hz)	14.7	1.2	13.7	1.1	0.04*
Peak vertical force (bw)	2.7	0.3	2.5	0.3	0.03*
Peak horizontal force (bw)	14.5	3.6	14.1	2.5	0.76
Horizontal impulse (bw × s)	2.6	0.3	2.9	0.2	<0.01**
Maximum rearfoot motion (°)	8.5	2.6	10.9	3.1	0.04*
Peak pressure lateral heel (kPa)	735	294	743	189	0.93
Peak pressure medial heel (kPa)	657	377	719	263	0.62
Peak pressure lateral midfoot (kPa)	343	117	263	57	0.03*
Peak pressure metatarsal head V (kPa)	422	236	480	148	0.45*
Peak pressure metatarsal head III (kPa)	673	203	525	150	0.04*
Peak pressure metatarsal head I (kPa)	713	203	676	191	0.71
Peak pressure hallux (kPa)	581	224	533	266	0.68

**highly significant at *P* < 0.01, *significant at *P* < 0.05.

**Table 5:** VW shod running with inserted valgus wedges

**Variables**	**Semiprofessional group mean**	**SD**	**Amateur group mean**	**SD**	***P *** **value**
Loading rate (bw/s)	97.1	45.5	62.2	12.2	0.01*
Peak tibial acceleration (g)	7.8	3.0	5	2.2	<0.01**
Median power frequency (Hz)	13.9	0.9	12.9	1.3	0.02*
Peak vertical force (bw)	2.7	0.3	2.5	0.3	0.01*
Peak horizontal force (bw)	13.8	3.5	13.2	2.4	0.58
Horizontal impulse (bw × s)	2.6	0.3	2.8	0.3	0.09
Maximum rearfoot motion (°)	9.0	2.9	11.5	2.8	0.03*
Peak pressure lateral heel (kPa)	690	349	513	183	0.11
Peak pressure medial heel (kPa)	516	257	617	229	0.28
Peak pressure lateral midfoot (kPa)	407	177	355	53	<0.05*
Peak pressure metatarsal head V (kPa)	377	193	433	88	0.34
Peak pressure metatarsal head III (kPa)	627	244	462	157	0.04*
Peak pressure metatarsal head I (kPa)	626	172	646	188	0.83
Peak pressure hallux (kPa)	528	271	518	241	0.94

**highly significant at *P* < 0.01, *significant at *P* < 0.05.

The reduced shock absorption for the semiprofessional subjects is also demonstrated by an increased peak tibial acceleration (NW: +32%, p < 0.05, VW: +56%, p < 0.01) when compared to the amateur soccer players. Maximum rearfoot motion is about 22% lower in the semiprofessional soccer players compared to the amateur group in both shoe conditions. In the second part of the stance phase, peak vertical force is increased (NW/VW: +8%, p < 0.05) while the horizontal impulse is reduced (NW: -10%, p < 0.05; VW: -7%, p = 0.08) in the semiprofessional subjects. Additionally, we found increased peak plantar pressures under the lateral midfoot (NW: + 30%, p < 0.05, VW: +15%, p < 0.05) and the third metatarsal head (NW: + 30%, p < 0.05, VW: +36%) in the semiprofessional group for running at the same speed (3.3 m/s).

## Discussion

Purpose of the present study was to investigate biomechanical manifestations of soccer players with increased alpha angles during running, especially the rearfoot motion, which was not been analyzed before. To our best knowledge, this was the first gait analysis of predominantly asymptomatic athletes which had radiologically increased alpha angles. Despite no apparent differences in foot structure, the semiprofessional group experience higher impact forces and load transfer at initial ground contact as compared to the subjects of the amateur group. Increases in the median power frequency, maximum vertical ground reaction force rate, and peak tibial acceleration indicate the semiprofessional group’s lower limb to be a more rigid structure during ground contact in running when compared to the amateur subjects. The lower rearfoot motion in the semiprofessional group also contributes to increased shocks of the body during ground contact. A more lateral placement of the foot, causing higher peak pressures under the lateral heel and third metatarsal head, may explain the more rigid foot structure with less subtalar angular joint motion.

Although hip motion during running was not measured, the present data allow us to assume that the oscillation of the center of mass is different between the investigated groups. In the semiprofessional group, increased vertical forces are accompanied by reduced horizontal forces which both are related to a more accentuated upward-downward movement of the center of mass. Compared to non-symptomatic subjects, FAI-patients are restricted in hip flexion during squatting which may be caused by hip pain [25]. The half of the semiprofessional subjects showed clinically a varus deviation of the lower limb. In a previous study, our study group found a correlation between increased alpha angles and deviation of the mechanical leg axis [26]. The mechanical axis deviation can have effects on the kinematics on gait. To compensate limitations in hip flexion which was found in FAI patients during walking, increasing vertical oscillation of the center of mass could be used to counteract the restrictions in sagittal plane hip motion [17]. By applying this strategy, soccer players with increased alpha angles may achieve the same step length but with higher lower extremity loading as well as, obviously, higher energy consumption due to increases in mechanical work. Obviously, all compensatory mechanisms become manifest as a more rigid lower limb structure during the entire stance phase.

It can be concluded that limitations in range of motion (ROM) which were observed in the studies of Kennedy et al. [17] and Lamontagne et al. [25] seem to be compensated by readjustments of the locomotor system. However, restrictions in ROM combined with reduced shock absorption capacity constitute interlocking risk factors for joint disease. Especially in running-related activities, such as in many team sports, the need to be competitive has to be discussed critically according to the aftereffects, especially the pathomechanism of osteoarthrosis.

Consistent with previous findings, our study suggests that specific characteristics of FAI, for instance functional constraints in ROM, can be diagnosed by biomechanical screening. Limitations in hip ROM, reduced shock attenuation capacity and accentuated vertical movement of the center of mass should be considered as functional indicators of FAI.

## Conclusions

Future research is needed to identify characteristic features which display the different stages of FAI process. A challenge for future research is to develop physical training programs for preventing FAI or decelerating the FAI process. These findings support the need for a screening program for competitive soccer players. In cases of a conspicuous gait analysis, FAI must be ruled out by further diagnostic tests.

## Abbreviations

FAI: Femoroacetabular impingement; FOV: Field of view; MRI: Magnetic resonance imaging; NW: No wedges; TE: Echo time; TR: Repetition time; ROM: Range of motion; VW: Valgus wedges.

## Competing interests

The authors declare that they have no competing interests.

## Authors’ contributions

ML, AF, CA, EMH and MH conceived and designed the study. ML, PAW, CL and LVvE performed the MRI measurements. ML, CvSP, PAW, KD, CA, EMH and MH are involved in the execution of the study and the writing of this manuscript. All authors read and approved the final manuscript.

## Pre-publication history

The pre-publication history for this paper can be accessed here:

http://www.biomedcentral.com/1471-2474/15/88/prepub
